# Biweekly cetuximab and irinotecan in advanced colorectal cancer patients progressing after at least one previous line of chemotherapy: results of a phase II single institution trial

**DOI:** 10.1038/sj.bjc.6604530

**Published:** 2008-07-29

**Authors:** P Martín-Martorell, S Roselló, E Rodríguez-Braun, I Chirivella, A Bosch, A Cervantes

**Affiliations:** 1 Department of Haematology and Medical Oncology, Hospital Clínico Universitario, University of Valencia, Valencia, Spain

**Keywords:** metastatic colorectal cancer, irinotecan, cetuximab, biweekly schedule

## Abstract

This is a phase II institutional exploratory trial of biweekly irinotecan and cetuximab administration regimen in metastatic colorectal cancer patients progressing to at least one previous chemotherapy line. A total of 40 patients were treated between November 2005 and November 2007 with irinotecan 180 mg m^−2^ and cetuximab 500 mg m^−2^ q2w (every 2 weeks), in every 21-day cycles, until unacceptable toxicity or progressive disease. An overall response rate of 22.5% was obtained (two complete and seven partial responses). The disease control rate was 60%. The time to progression was 3.4 months and the overall survival was 8 months. The toxicity compared very favourably to weekly cetuximab combination schedules. Grade 3/4 adverse effects were observed in 12 patients. Overall, our results turn up very similar both in terms of toxicity and efficacy to those obtained by weekly and biweekly administration regimens.

Cetuximab is an epidermal growth factor receptor (EGFR)-directed IgG1 chimeric monoclonal antibody showing antitumour activity in the treatment of advanced colorectal cancer. Cetuximab binds to the extracellular domain of EGFR when it is in the inactive configuration, competes for receptor binding by occluding the ligand-binding region, and thereby blocks ligand-induced EGFR tyrosine kinase activation (Ciardello and Tortora, 2008)

In a randomised phase II trial comparing a combination of weekly cetuximab and biweekly irinotecan with weekly cetuximab monotherapy, this monoclonal antibody proved to have consistent antitumour activity in patients with advanced colorectal cancer refractory to irinotecan (Cunningham *et al*, 2004). This study led to the approval of cetuximab by the regulatory authorities for the treatment of irinotecan refractory metastatic colorectal cancer. Other trials have shown that cetuximab improves survival over best supportive care alone (Jonker *et al*, 2007) and might offer some advantage in patients receiving first or second line therapy (Van Cutsem *et al*, 2007, Pessino *et al*, 2008, Sobrero *et al*, 2008). Moreover, it has also proved to be active in combination with oxaliplatin-based regimens, both in the first (Tabernero *et al*, 2007) and successive lines of treatment (Souglakos *et al*, 2007)

In all those studies, cetuximab was administered weekly with an initial intravenous infusion of 400 mg m^−2^ on day 1 with subsequent weekly doses of 250 mg m^−2^. Although this regimen is undoubtedly active, the weekly administration of cetuximab is out of step with administration of the chemotherapy regimens with which cetuximab is commonly combined. Irinotecan is often administered at a dose of 180 mg m^−2^ every 2 weeks. Similarly, in the first-line setting, combinations of infusional 5-fluorouracil (5-FU)/folinic acid plus irinotecan (FOLFIRI) or oxaliplatin are frequently administered on an every 2 weeks basis. The option to synchronise the administration of cetuximab and concomitant chemotherapy would reduce the impact of treatment administration on patients' lives and simplify treatment administration for health-care workers. It is also reasonable to assume that a simplified schedule may reduce the costs associated with cetuximab administration.

In a preliminary reported phase I trial, it was shown that cetuximab can be safely administered at 500 mg m^−2^ every 2 weeks, with similar pharmacokinetic and pharmacodynamic behaviour compared with the weekly schedule (Tabernero *et al*, 2006). The biweekly dosing may facilitate the administration of this drug, by making this therapy more convenient for patients (Tabernero *et al*, 2008). This publication reports on a phase II trial designed to explore the antitumour activity of combined irinotecan and cetuximab, both administered in a biweekly fashion.

## Materials and methods

### Study design

This was an institutional prospective, single-arm phase II trial exploring the antitumour activity of biweekly administration of cetuximab and irinotecan in patients with metastatic colorectal cancer who had progressed to at least one previous line of chemotherapy for advanced disease. The primary end point was response rate. Secondary end points were toxicity, time to progression, duration of response and overall survival. Patients were recruited between November 2005 and November 2007. Analysis of data took place on January 2008. All patients gave their informed consent before treatment and the trial was performed according to the Institutional Review Board.

### Selection of patients

Eligibility criteria were histologically confirmed colorectal adenocarcinoma, with progressive metastatic disease to at least one previous line of chemotherapy. Patients had to be at least 18 years or older, have a performance status of 0–2, adequate bone marrow reserve (Hb ⩾8.0 g dl^−1^, neutrophil count ⩾1.5 × 10^9^/l, platelet count ⩾100 × 10^9^/l), hepatic and renal function (total bilirubin <1.5 UNL, ASAT and ALAT <2.0 UNL and serum creatinine <2 mg dl^−1^). Epidermal growth factor receptor immunohistochemistry and k-ras status were not required in the eligibility criteria.

### Therapy

Eligible patients were treated with cetuximab 500 mg m^−2^ intravenous infusion on day 1 (during 2 h on the first infusion and during 1 h on subsequent cycles if no adverse reaction had occurred on the previous administration), followed by irinotecan 180 mg m^−2^ intravenous infusion on day 1 (during 30 min in all cycles). Before cetuximab, all patients received dexchlorphenamine maleate at a dose of 5 mg intravenously. Antiemetic prophylaxis with dexamethasone and ondansetron was given before irinotecan. Patients were evaluated with blood count, complete serum biochemistry and CEA before day 1 initially. If no relevant toxicity occurred, this was then done every other cycle. Treatment was continued until documented disease progression or unacceptable toxicity, whichever occurred first.

### Evaluation of safety and response

Evaluation of disease was carried out according to RECIST criteria (Therasse *et al*, 2000) every 3–5 cycles. Toxicity was evaluated according to NCI-CTCAE (version 3.0), before every treatment of the first three cycles, and every other cycle thereafter if no relevant toxicity had appeared. Irinotecan dosage was reduced by 25% if ⩾grade 3 diarrhoea was observed. Cetuximab administration was delayed if cutaneous toxicity ⩾grade 3 was observed and restarted when it had reduced to grade 2.

### Statistical analysis

Times to event variables were calculated according to Kaplan–Meier methods using StatSoft (version 6). Descriptive variables of patient characteristics and toxicity were calculated directly from the database. Time to tumour progression (TTP) was defined as the time to documented progression from the start of the treatment, duration of response (DR) as the time from first objective response to documented progression and overall survival (OS) was considered from the start of treatment to date of data analysis or date of loss from follow-up for patients alive. Patients without disease progression who discontinued the study for any reason were censored at the last on study tumour assessment date. All efficacy and safety analyses were evaluated at an exploratory level.

## Results

Between November 2005 and November 2007, a total of 40 patients were recruited. Patient characteristics are shown in Table 1. Median age at diagnosis was 61 years. The median performance status at the start of treatment was 1. Patients had received a median of one previous chemotherapy line for advanced disease, but 27.5% had two previous lines and 20% got this therapy as fourth or further line. The median number of metastatic sites was 2. Almost all patients were pretreated with oxaliplatin- and fluoropyrimidine-based combinations. Half of them received previous treatment with bevacizumab and 25% received irinotecan-based therapies. The median follow-up time for the patients alive was 5 months (range: 4–24.5). A total of 322 treatment cycles were administered, which amounts to a median of 7 cycles per patient (range: 2–29).

### Efficacy

**Figure 1:**
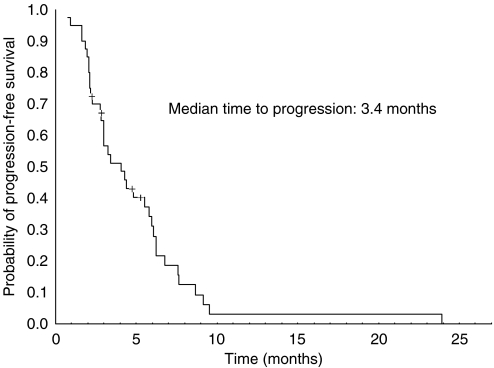
Progression-free survival Kaplan–Meier curve. The median time to progression was 3.4 months (range: 0.7–23.9). Thirty-six out of 40 patients had progressed at the time of analysis.

**Figure 2:**
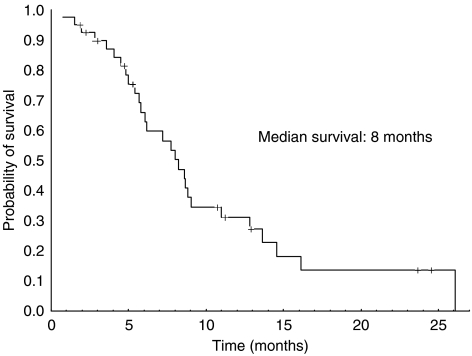
Overall survival Kaplan–Meier curve. The median overall survival time from the start of treatment was 8 months (range: 0.7–26.1). Twenty-eight patients out of the 40 registered in the trial had died at the time of analysis.

A total of 39 patients were assessed for response. There was an overall response rate of 22.5% (two complete and seven partial responses, CI 95%: 9.6–35.4%). Stable disease was observed in 15 cases (37.5%). The disease control rate was 60% (CI 95%: 44.9–75.1%). Progressive disease was observed in 15 patients. One patient was not assessable because she died before evaluation. The median TTP was 3.4 months (range: 0.7–23.9). Figure 1 shows the progression-free survival curve. The median OS from the start of treatment was 8 months (range: 0.7–26.1). Figure 2 shows the Kaplan–Meier OS curve. Eight out of the nine responding patients had progressed at the time of analysis. The median duration of response was 5.0 months (range: 2–20).

### Safety

The biweekly administration of cetuximab and irinotecan proved to be tolerable. Considering maximum reported toxicity by patient, the only grade 4 observed event was diarrhoea in two patients (5%). Two more patients suffered from grade 3 diarrhoea (5%). Grade 3 skin rash was seen in three patients (7.5%). Grade 3 anaemia and neutropoenia were reported in one (2.5%) and three (7.5%) cases, respectively. Only one case of grade 3 nausea was seen. Neither grade 4 myelosuppression nor severe infusional anaphylactic reactions were observed. Concerning grade 2 events, paronychia was reported in 30% of patients, alopecia in 7.5%, skin rash in 17.5% and asthenia in 15%. No treatment related deaths occurred.

## Discussion

The aim of this exploratory trial was to investigate the antitumour activity of the combination of irinotecan and cetuximab given in a biweekly fashion, as well as its safety profile with respect to the standard weekly regimen used in most trials with cetuximab. The efficacy data obtained in the different studies of irinotecan and cetuximab given weekly or biweekly are listed in Table 2. The response rate seen in this trial is very similar to that observed with weekly cetuximab administration in the BOND trial (Cunningham *et al*, 2004). Also time to progression (3.4 *vs* 4.1 months) and overall survival (8 *vs* 8.6 months) illustrate the similarities of both schedules. Our results are also comparable to those obtained in a large confirmatory trial accruing more than 1000 patients (Wilke *et al*, 2006). Safety between the different schedules is compared in Table 3. In terms of toxicity, the biweekly regimen also proved to be tolerable. Grade 3 or 4 events were mainly observed only in eight (20) and two (5%) of our patients, respectively. Pfeiffer *et al* reported very similar results using a biweekly cetuximab and irinotecan regimen. Their results using the biweekly schedule were comparable to those of a 65 patients historical cohort receiving the weekly schedule at the same participating institutions.

Different phase I and II trials have studied the pharmacokinetic and pharmacodynamic behaviour of cetuximab in different dosing schedules. Fairly predictable linear pharmacokinetic has been demonstrated for cetuximab. Multiple dose studies have demonstrated that the pharmacokinetic parameters – CL, AUC, *t*_1/2_ and volume of distribution at steady state (*V*_ss_) – are similar after single and multiple doses of cetuximab at the approved dosing regimen. The AUC also shows a linear relationship to the dose and frequency of administration (Humblet *et al*, 2005). The data regarding pharmcokinetic and pharmacodynamic behaviour of cetuximab at different dosages and frequency schedules support the feasibility of biweekly cetuximab administration in combination with irinotecan (Tabernero *et al*, 2006).

Furthermore, the different trials using the simplified biweekly administration have all yielded very consistent results, both with regard to the TTP and OS, as well as to the response rate. Both our results and those recently published by Pfeiffer *et al*, show very similar efficacy data compared to the weekly schedule. Administering irinotecan and cetuximab together every 2 weeks would render conveniency both for patients and for the health resources, without compromising efficacy or having a deleterious effect on toxicity. It therefore appears as a very reasonable strategy that would be worth testing in future trials.

## Figures and Tables

**Table 1:** Baseline characteristics of patients

**Characteristic**	
Age – median (range) (years)	62 (33–78)
	
*Sex – n* (%)	
Male	19 (47)
Female	21 (53)
	
*Performance status – n* (%)	
0	9 (22.5)
1	25 (62.5)
2	6 (15)
	
*No. of metastatic localizations at start of treatment – n* (%)	
1	8 (20)
2	16 (40)
3	12 (30)
4	2 (5)
5	2 (5)
	
*No. of previous treatments for mCRC – n* (%)	
1	21 (52.5)
2	11 (27.5)
3 or more	8 (20)
	
*Prior therapy for mCRC – n* (%)	
Oxaliplatin	39 (97.5)
Fluoropyrimidine	40 (100)
Irinotecan	10 (25)
Bevacizumab	20 (50)

**Table 2:** Efficacy of cetuximab and irinotecan in metastatic colorectal cancer patients refractory to irinotecan-based therapy: comparison between weekly and biweekly combination regimens

	**Weekly regimens**			**Biweekly regimens**	
	**Cunningham *et al* (2004) (*N*=329)**	**Wilke *et al* (2006) (*N*=1147)**	**Pfeiffer *et al* (2008) (*N*=65)**	**Pfeiffer *et al* (2008) (*N*=74)**	**This study (*N*=40)**
RR (%)	22.9	20	20	25.7	22.5
TTP (months)	4.1	NR	5.4	4.8	3.4
OS (months)	8.6	9.2	10.4	9.8	8

NR=not reported; OS=overall survival; TTP=time to progression.

**Table 3:** Grade 3 or 4 adverse events reported in the treatment of metastatic colorectal cancer patients with irinotecan and cetuximab combination: comparison between the weekly and biweekly administration schedules

	**Weekly regimens (%)**			**Biweekly regimens (%)**	
**Adverse event**	**Cunningham *et al* (2004) (*N*=327)**	**Wilke *et al* (2006) (*N*=1147)**	**Pfeiffer *et al* (2008) (*N*=65)**	**Pfeiffer *et al* (2008) (*N*=74)**	**This study (*N*=40)**
Diarrhoea	21	20	10	9	10
Skin or nail toxicity	9	19	11	8	7.5
Fatigue/asthenia	14	8	8	4	0
Neutropoenia	9	10	4	7	7.5
